# Brugada Electrocardiogram Pattern Induced by Recreational Delta-8-Tetrahydrocannabinol (THC): A Case Report

**DOI:** 10.7759/cureus.19058

**Published:** 2021-10-26

**Authors:** Noah Y Jo, Chu-Chiao Chu, Bryan C Ramsey

**Affiliations:** 1 Internal Medicine, Brooke Army Medical Center, Fort Sam Houston, USA; 2 Cardiology, Brooke Army Medical Center, Fort Sam Houston, USA; 3 Interventional Cardiology, Brooke Army Medical Center, Fort Sam Houston, USA

**Keywords:** brugada syndrome, brugada, recreational, delta-8, tetrahydrocannabinol, bep, brugada electrocardiogram pattern

## Abstract

Brugada electrocardiogram (ECG) pattern describes a characteristic right bundle branch block (RBBB) appearance with persistent ST-segment elevation in precordial leads V1 to V3, often associated with Brugada syndrome, a genetic sodium channelopathy, in the absence of ischemic or structural heart disease. Known triggers such as fever, electrolyte abnormalities, medications, or recreational drugs may elicit such an ECG pattern without a clear clinical significance yet creating a dilemma for clinicians providing care in the urgent setting. We present a case of reversible Brugada electrocardiogram pattern (BEP) after recreational use of delta-8-tetrahydrocannabinol (THC) and explore the need for further research on the safety of such an over-the-counter supplement.

## Introduction

Marijuana is the most commonly used illicit substance in the United States [1] with varying rates of legalizations across the country for both recreational and medical use. Marijuana is developed from the plant cannabis with over 140 compounds of phytocannabinoids and terpenoids, which interact with the endocannabinoid system via cannabinoid receptor 1 (CB1R) and cannabinoid receptor 2 (CB2R) that are widely dispersed in the human body [2]. Of these compounds, tetrahydrocannabinol (THC) and cannabidiol (CBD) are the most studied compounds with contrasting properties [2]. While THC is primarily associated with inducing psychotropic “high” effects, the anxiolytic effects of CBD have generated numerous research studies, studying its potential therapeutic properties [2]. Varying degree of exposure to cannabinoids produces different cardiovascular effects, including but not limited to, tachycardia, bradycardia, hypotension, hypertension, arrhythmias, and shock [3,4]. There has been a rising number of case reports describing the association between cannabis consumption and transient Brugada electrocardiogram pattern (BEP), with the first case report of self-limiting electrocardiogram (ECG) changes that mimic BEP after cannabis intoxication in 2007 [5]. We present a case describing a progression of baseline type III to type I BEP after consumption of recreational delta-8 THC gummy.

## Case presentation

**Figure 1:**
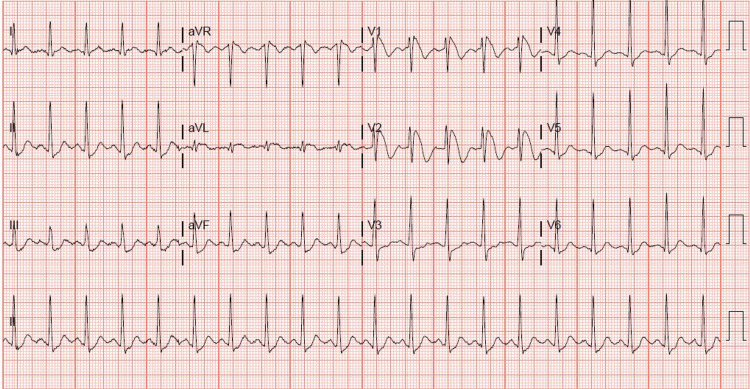
Admission 12-lead ECG with Brugada type 1 pattern.

**Figure 2:**
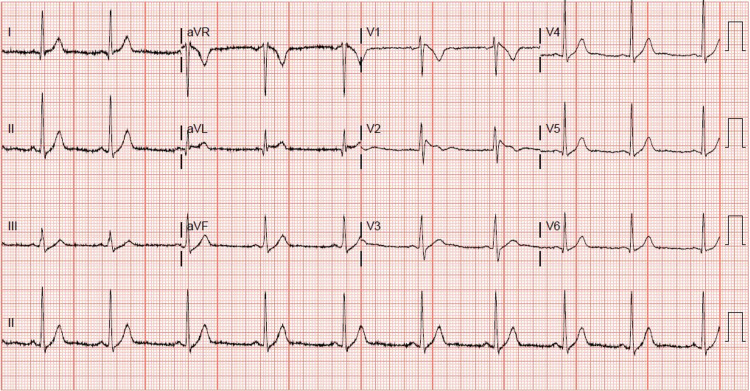
Baseline 12-lead ECG with Brugada type 3 pattern.

**Figure 3:**
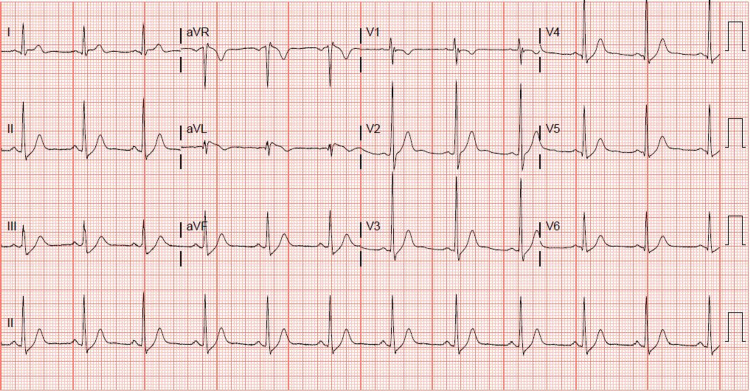
One-month outpatient follow-up 12-lead ECG without Brugada type features.

A 31-year-old, otherwise healthy Hispanic male, presented to our institution with sudden onset of chest pain 20 minutes after ingesting recreational delta-8 THC gummy. The chest pain was non-specific, as it was substernal, but not exertional, and not relieved with rest or nitroglycerin. Associated pallor and dyspnea were reported with his chest pain. A code STEMI (ST-segment elevation myocardial infarction) was called on presentation, given the noted ST elevation on 12-lead ECG. His ECG revealed coved ST elevations in V1 and V2, consistent with Brugada type 1 pattern (Figure 1), while his baseline ECG findings two months prior to presentation suggested Brugada type 3 pattern (Figure 2). Given his new ECG changes and unremitting chest pain, he was taken to the catheterization lab urgently for further evaluation. Left heart catheterization was unremarkable with no significant stenosis or evidence of any obstructive coronary artery disease. Left ventriculography showed normal left ventricular ejection fraction at 65-70% without regional wall motion abnormalities and normal left ventricular end-diastolic pressure. Initial screening labs were unremarkable, with no electrolyte abnormalities, undetectable serial troponins, and low brain natriuretic peptide levels. An echocardiogram again confirmed preserved left ventricular ejection fraction without wall motion abnormalities. ECG 16 hours post-delta-8 THC ingestion returned to baseline Brugada type 3 pattern the following morning, and his Brugada pattern ultimately resolved upon outpatient one-month follow-up (Figure 3).

## Discussion

Brugada syndrome is an autosomal dominant, genetic arrhythmia syndrome with known channelopathy defects, most commonly in the cardiac sodium channels (but also with known genetic abnormalities in the calcium and potassium channels), and varying penetrance [6,7]. The first description of the Brugada syndrome was reported in 1992, presenting eight patients with repeated aborts of sudden cardiac deaths (SCD) with ECG findings showing a right bundle branch block, persistent ST-segment elevation in precordial leads V1 to V2-V3, and normal QT interval in the absence of electrolyte abnormalities, ischemic, or structural heart disease [8]. The most recent definition of Brugada syndrome describes ECG findings showing type 1 morphology that occurs either spontaneously or after provocative testing with pharmacologic agents [6]. Brugada electrocardiogram pattern (BEP) is differentiated from Brugada syndrome with its characteristic ECG findings with the absence of clinical symptoms including arrhythmia-related symptoms, sustained ventricular arrhythmias, or SCD (including a family history of SCD younger than age 45 or with BEP type 1 in relatives) [9]. BEP is commonly associated with the intoxication of illicit substances, metabolic derangements, electrolyte abnormalities, myocardial and pericardial disease, and ECG modulation [10]. While Brugada syndrome is typically considered an inherited disease, an increasing number of case reports show the association of BEP with cannabis intoxication [5,7,9,11-12]. Our case highlights the novel association of BEP with a specific content of cannabis (delta-8 THC).

The exact mechanism of BEP remains speculative, as it has previously been attributed to either defect in cardiac ion channels resulting in depolarization/repolarization or abnormal expression of neural crest cells [13]. Our patient demonstrated reversible BEP after ingestion of delta-8 THC, which raises concerns about the safety profile of delta-8 THC. Although delta-9 THC has been known as the main psychoactive compound of marijuana [14,15], delta-8 THC's effects have been less studied, and its exact interaction with the cannabinoid receptors on cardiac ion channels has not been elucidated. Other illicit substances, such as cocaine, have been studied to exert sodium-channel blocking effects that cause higher intracellular calcium levels, and thereby increase the risk of ventricular arrhythmias [10]. However, such a mechanism has not been studied for delta-8 THC. Furthermore, it is unclear which patients are predisposed to developing BEP or Brugada syndrome when exposed to the contents of cannabis. A prior study by Hermida et al. looked at the prevalence of drug-induced BEP via provocative drug tests using sodium channel blockage agents. The study noted that of the five of 1,000 subjects who demonstrated drug-induced BEP, none had the SCN5A mutation that is commonly associated with Brugada syndrome [16]. Due to the increasing legalization and availability of marijuana in its different forms, further research is warranted to elucidate the harmful effects of the substance.

## Conclusions

This case report presents an association between the use of marijuana via the form of delta-8 THC gummy and BEP. The purpose of this case presentation was to raise awareness about the increasing number of cases of Brugada syndrome and BEP associated with the different components of marijuana use (both legal and illegal), thus highlighting the need for further research and regulation of marijuana and its contents to reduce cardiac risk to the consumer population.
